# School based screening for tuberculosis infection in Norway: comparison of positive tuberculin skin test with interferon-gamma release assay

**DOI:** 10.1186/1471-2334-8-140

**Published:** 2008-10-17

**Authors:** Brita Askeland Winje, Fredrik Oftung, Gro Ellen Korsvold, Turid Mannsåker, Ingvild Nesthus Ly, Ingunn Harstad, Anne Margarita Dyrhol-Riise, Einar Heldal

**Affiliations:** 1 Division of Infectious Disease Control, Norwegian Institute of Public Health, 0403 Oslo, Norway; 2 Department of Medicine, Ullevaal University Hospital, Kirkeveien 166, 0407 Oslo, Norway; 3 Faculty of Medicine, Norwegian University of Science and Technology, 7941 Trondheim, Norway; 4 Department of Medicine, Haukeland University Hospital, 5021 Bergen, Norway

## Abstract

**Background:**

In Norway, screening for tuberculosis infection by tuberculin skin test (TST) has been offered for several decades to all children in 9th grade of school, prior to BCG-vaccination. The incidence of tuberculosis in Norway is low and infection with *M. tuberculosis *is considered rare. QuantiFERON^®^TB Gold (QFT) is a new and specific blood test for tuberculosis infection. So far, there have been few reports of QFT used in screening of predominantly unexposed, healthy, TST-positive children, including first and second generation immigrants. In order to evaluate the current TST screening and BCG-vaccination programme we aimed to (1) measure the prevalence of QFT positivity among TST positive children identified in the school based screening, and (2) measure the association between demographic and clinical risk factors for tuberculosis infection and QFT positivity.

**Methods:**

This cross-sectional multi-centre study was conducted during the school year 2005–6 and the TST positive children were recruited from seven public hospitals covering rural and urban areas in Norway. Participation included a QFT test and a questionnaire regarding demographic and clinical risk factors for latent infection. All positive QFT results were confirmed by re-analysis of the same plasma sample. If the confirmatory test was negative the result was reported as non-conclusive and the participant was offered a new test.

**Results:**

Among 511 TST positive children only 9% (44) had a confirmed positive QFT result. QFT positivity was associated with larger TST induration, origin outside Western countries and known exposure to tuberculosis. Most children (79%) had TST reactions in the range of 6–14 mm; 5% of these were QFT positive. Discrepant results between the tests were common even for TST reactions above 15 mm, as only 22 % had a positive QFT.

**Conclusion:**

The results support the assumption that factors other than tuberculosis infection are widely contributing to positive TST results in this group and indicate the improved specificity of QFT for latent tuberculosis. Our study suggests a very low prevalence of latent tuberculosis infection among 9th grade school children in Norway. The result will inform the discussion in Norway of the usefulness of the current TST screening and BCG-policy.

## Background

The incidence of tuberculosis in Norway is generally low (6.3/100 000 population in 2006), but high among immigrants from countries where tuberculosis is endemic [1]. Although the tuberculin skin test (TST) has low specificity, it is still the major tool for detecting tuberculosis infection. Screening for tuberculosis infection by TST has in Norway been offered to all children in the 9th grade of school (age 14–15) for several decades. Historically there have been three objectives of this screening: (1) to measure the transmission rate of tuberculosis infection in the population, (2) to identify cases with latent tuberculosis infection for preventive treatment, and (3) to ensure that only tuberculin negative children receive Bacillus Calmette-Guérin (BCG) vaccine, which is offered in the national vaccination programme to all previously unvaccinated, TST negative children at the age of 14 years. Children born in Norway of parents from high prevalence countries and a limited number of other children assumed to be at risk are offered BCG-vaccination at birth or on other occasions [2].

Each year, this screening results in several hundred children with a positive TST (defined as => 6 mm) being referred to hospitals for medical evaluation, chest X-ray and a three year follow up procedure. This requires large resources and causes concern among the affected families. No case of tuberculosis disease has been identified through this screening for many years. We suspect that previous BCG-vaccination or infection with non-tuberculosis mycobacteria (NTM) are the causes of most TST reactions as few of the children have any known exposure to tuberculosis and the transmission rate in Norway, based on molecular epidemiology, is known to be very low [3,4].

New *in vitro *assays based on cellular production of interferon-gamma (IFN-γ) in response to the *M. tuberculosis *specific antigens ESAT-6, CFP10, and TB7.7 have been developed. These protein antigens are absent in all vaccine strains of *M. bovis*-BCG and most NTM, except *M. marinum, M. szulgai, and M. kansasii *[5,6]. In contrast to TST, these tests can therefore distinguish *M. tuberculosis *infection from infections with NTM or previous BCG-vaccination. The assays seem to be accurate and valid diagnostic tools for both active and latent tuberculosis infection with high specificity [6-10]. Among the commercially available and regulatory approved IFN-γ release assays, the QuantiFERON^®^TB Gold (QFT) test offers both logistic and economical advantages which make it suitable for mass screening. QFT has now been evaluated and used in different contexts. So far, there have been few reports of QFT used in screening of predominantly unexposed and healthy TST positive children, including first and second generation immigrants [6,8,11,12].

The policy of universal TST screening and BCG-vaccination of school children in Norway is currently under discussion. In order to evaluate the current TST screening and BCG-vaccination programme and inform the discussion on any future targeted screening approach, we aimed to (1) measure the prevalence of QFT positivity among TST positive children identified in the school based screening and (2) measure the association between demographic and clinical risk factors for tuberculosis infection and QFT positivity.

## Methods

In Norway, all children in the 9th grade of school (age 14–15) are offered TST screening. TST with purified protein derivative (PPD) RT 23 (2 TU) from Statens Serum Institute, Denmark, is applied according to the Mantoux method and read by public health nurses in the schools. This antigen is comparable with 5 TU of PPD in the United States and Canada [13]. A test is considered positive if the induration is => 6 mm after 72 hours. Children with a positive TST are referred to the public hospital serving the area. In this cross-sectional study in the school year 2005–6, we aimed to include all 9th grade children referred to seven hospitals serving both rural and urban areas in Norway. After the parents or guardians had given written informed consent, a QFT test was obtained and a questionnaire regarding demographic and clinical risk factors for latent infection, including data on exposure, was completed. Regardless of participation in the study, the nationally recommended follow up procedures were followed.

Statistics Norway provided the number and demographic characteristics of all children born in 1991 (proxy for 9th graders) living in the geographical regions covered by the seven hospitals. The Regional Ethics Committee for Medical Research recommended the study (S-05160) and permission was given from the Norwegian Data Inspectorate (23147).

The QuantiFERON^®^TB Gold in-tube-test (Cellestis Ltd, Victoria, Australia) was used. One ml of venous blood was drawn into each of two pre-coated tubes with and without synthetic peptide antigens respectively. A mitogen positive control was not included since this was not available in this first version of the test. The samples were processed and stored in accordance with the manufacturer's instructions at the local hospital before they were transported to the Norwegian Institute of Public Health, where harvested plasma was subjected to Enzyme-Linked Immunosorbent Assay (ELISA) analysis, including IFN-γ standard for quantification. The quality of all laboratory analysis and calculation of the results was controlled by the accompanying QFT analysis software. A sample was considered positive if exceeding the standard cut-off value at 0.35 IU IFN-γ/ml. All positive results were confirmed by re-analysis of the same plasma sample before they were reported as positive. If the confirmatory test was negative, the QFT result was reported as non-conclusive and the participant was offered a new test.

Participating hospitals entered data into Excel 2003 in a standardized format and the data were later validated and merged at the Norwegian Institute of Public Health. Exact confidence intervals were calculated for proportions of single samples. Statistical analyses were performed in STATA 9.2 (Statacorp, Texas, 77845, USA).

For analysis the origin of children was grouped in three: (1) born in a Western country with parents of Western origin (further referred to as Western-born), (2) born in a Western country with one or both parents of non-Western origin (second generation immigrant), and (3) born in a non-Western country (first generation immigrant). Western countries were defined as USA, Australia, Japan, Canada, New Zealand, and European countries other than Turkey, the Balkan States and countries of the former Soviet Union. The size of the TST indurations was grouped into three: 6–9 mm, 10–14 mm and 15 mm or more. The presence of a scar was considered as evidence of BCG vaccination (scar/no scar) and self-reported known exposure to an infectious tuberculosis case was reported as exposure (yes/no). The accumulated duration of travels to non-Western countries was grouped in three: none, or </> 3 months.

Unconditional multivariate analysis (logistic regression) was performed with QFT as outcome variable. The independent variables; origin, gender, BCG-vaccination, exposure to tuberculosis and travel history were all included in a preliminary multivariable regression analysis. Spearman correlation coefficient was used to check for correlation among pairs of independent variables. We subtracted one variable at the time using the likelihood ratio test as elimination criterion (p < 0.05). The same approach was used to test the significance of the two-way interaction terms between the independent variables in the final model. This could not be run due to the low number of QFT positives. The odds ratios in the final model were used to measure the strength of association. A separate univariate regression analysis was performed to measure the strength of association between QFT positivity and travel history for Western-born and second generation immigrant children.

## Results

### Study population and characteristics

The 33456 children in the catchment areas of the seven participating hospitals constituted 53% of the children in Norway born in 1991. They had the same gender distribution as all children born in 1991, while children with immigrant parents were significantly overrepresented (Table 1). Among the Western-born children 86% were born in Norway, the remaining in other low-prevalence countries mainly in Scandinavia and in USA. A total of 531 TST positive children were referred and 519 (98%) consented to participate. Among 58 children with a positive first QFT-analysis, 16 (28%) had a negative result on the confirmatory analysis of the same plasma sample and the result was considered non-conclusive. Eight of them submitted a new blood sample for QFT testing, two were then positive and six negative. They are included in the study group based on the result of the second test. The remaining eight did not consent to repeat testing and were excluded from the study. Thus, altogether 511 children had a conclusive QFT result and formed the study group.

**Table 1:** Characteristics of source population and study group: number and prevalence of positive TST

**Variable**	**Population in nation born in 1991**	**Population in project area born in 1991**	**No. TST positives in project area**	**Prevalence of TST positives in project area**
	No.	No. (%)	No.	% (95% CI)
Total	63662	33456 (53)	531	1.6 (1.5 – 1.7)
Gender				
Male	32617	17127 (53)	286	1.7 (1.5 – 1.9)
Female	31045	16329 (53)	245	1.5 (1.3 – 1.7)
Origin of child				
Western-born	57775	29712 (51)	288	0.9 (0.9 – 1.1)
Second generation immigrant	2867	2052 (72)	111	5.4 (4.5 – 6.5)
First generation immigrant	3020	1692 (56)	132	7.8 (6.6 – 9.2)

Characteristics of children born in 1991 in nation and project area and 531 children referred because of positive tuberculin skin test result in the school based screening programme in Norway in the school year of 2005–6.

Among these 511 children, the median TST indurations for unvaccinated children were 10 mm (range 6–30 mm) and for previously BCG-vaccinated children 11 mm (range 6–45 mm). First- and second generation immigrant children significantly more often presented with a positive TST compared to Western-born children (Table 1). None of the children were diagnosed with tuberculosis disease during clinical evaluation or reported any previous history of tuberculosis.

### Prevalence of positive QFT result

**Figure 1:**
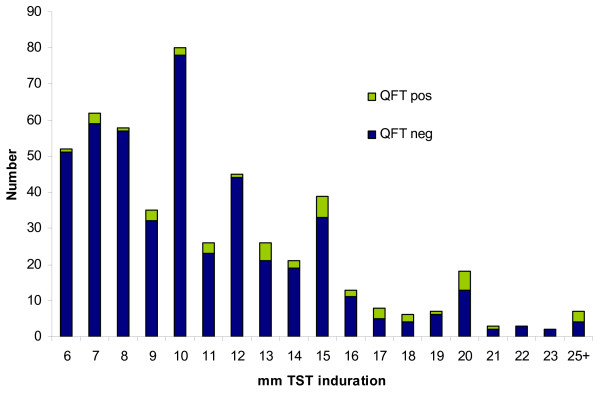
**Number of QuantiFERON^®^TB Gold positive results distributed by mm tuberculin skin test induration**. Distribution of QuantiFERON^®^TB Gold results among 511 tuberculin skin test positive children detected in the Norwegian school based screening programme in the school year of 2005–6. Tuberculin skin test indurations measured as 25 mm or more were grouped together as 25 mm+.

Only, 44 (9%) of the 511 TST positive children had a positive QFT result. Positive QFT tests were seen with all sizes of TST indurations (Figure 1), although the proportion increased with the size of the TST indurations in both genders and regardless of BCG vaccination and origin (Table 2). First generation immigrants significantly more often had a positive QFT test (18% of the TST positives), in contrast to 6% among both Western-born and second generation immigrants. Among the 16 Western-born children with positive QFT results, 15 were born in Norway. Seven of these children came from one coastal city in Southern Norway, which contributed to only 14% of the children in the study. Three of them came from the same school where additional nine children were identified with a positive TST, while the QFT turned out negative. They were all assessed for history of symptoms or clinical findings concurrent with *M. marinum, M. kansasii *or *M. szulgai *infection, but none reported such. Discrepant results were common even for TST reactions =>15 mm, of which only 22% had a positive QFT. Among the 83 participants with TST =>15 mm and negative QFT, 31 were previously unvaccinated.

**Table 2:** QuantiFERON^®^TB Gold positive results distributed by TST-induration, gender, BCG-status and origin

	**Total study group**			**TST 6–9 mm**		**TST 10–14 mm**		**TST =>15 mm**	
Variable	TST	QFT+		TST	QFT+	TST	QFT+	TST	QFT+
	No.	No. (%)	95% CI	No.	No. (%)	No.	No. (%)	No.	No. (%)
Study group	511	44 (9)	6.4 – 11.2	207	8 (4)	198	13 (7)	106	23 (22)
Gender									
*Male*	275	21 (8)	4.9 – 11.2	110	6 (6)	110	7 (6)	55	8 (15)
*Female*	236	23 (10)	6.4 – 14.0	97	2 (2)	88	6 (7)	51	15 (29)
Origin of child									
*Western-born*	280	16 (6)	3.4 – 8.9	131	6 (5)	110	7 (6)	39	3 (8)
*Second generation immigrant*	108	6 (6)	2.3 – 11.1	41	1 (2)	44	1 (2)	23	4 (17)
*First generation immigrant*	123	22 (18)	11.9 – 25.4	35	1 (3)	44	5 (11)	44	16 (36)
BCG-scar									
*Scar*	236	23 (10)	6.4 – 14.0	80	2 (3)	89	5 (6)	67	16 (24)
*No scar*	262	18 (7)	4.2 – 10.4	125	6 (5)	101	7 (7)	36	5 (14)
*Doubtful or no report*	13	3 (23)	6.2 – 50.9	2	0	8	1	3	2

Number of QuantiFERON^®^TB Gold positive results among 511 tuberculin skin test positive children detected in the Norwegian school based screening programme in the school year of 2005–6.

Among the 16 children with a non-conclusive first QFT-test, nine were Western-born, three were second and four were first generation immigrants and none reported exposure to tuberculosis. Their median TST indurations were 10 mm (range 6–20 mm). The IFN-γ levels in the first positive result varied between 0.35–0.5 IU IFN-γ/ml for seven, between 0.5–1.0 IU IFN-γ/ml for five and higher than 1 IU IFN-γ/ml for four of the children. For the two children with a positive result in a new test, the level of IFN-γ increased compared to the first test. Among the six children who had a negative result in a new test the level of IFN-γ in the first positive result differed widely.

### Predictors of a positive QFT result

Altogether 46% of the TST positive children had a BCG-scar, 84% among first generation immigrants, 89% among second generation immigrants and 13% among Western-born children (Table 2). Exposure to an infectious case of tuberculosis was reported by 27 children. Five out of seven children reporting exposure to a household contact had a positive QFT, while only two out of 20 if the source case was outside the household. Six out of seven with exposure and a positive QFT were immigrants.

Immigrant children reported staying in non-Western country prior to arrival to Norway with a median of 9 years (range 1 month-15 years). Second generation immigrant children (76%) reported travels to non-Western countries more often than Western-born children (34%), but the median accumulated duration of their travels was comparable (6 weeks). Only origin and exposure to tuberculosis were associated with QFT positivity (Table 3). Both BCG-vaccination and travel history were correlated to origin (Spearman's rho 0.735 and 0.663 respectively) and did not fit into the same model. Travel was not significantly associated with a positive QFT for Western-born or second generation immigrant children in univariate regression analysis (data not shown).

**Table 3:** Associations between background variables and positive QuantiFERON^®^TB Gold test

**Variable**	**Total**	**QFT+**	**a OR**	**p-value for variable**
**Origin of child**				0.001
***Western-born***	280	16	ref	
***Second generation immigrant ***	108	6	0.9 (0.3 – 2.4)	
***First generation immigrant ***	123	22	3.3 (1.6 – 6.2)	
**Exposure**				0.034
***No***	478	37	ref	
***Yes***	27	7	2.9 (1.1 – 7.6)	

Results from multivariate regression analysis with positive QuantiFERON^®^TB Gold test as outcome variable among 511 tuberculin skin test positive children detected in the Norwegian school based screening programme in 2005–6. Final model; 505 observations, p-value < 0.001 and Log likelihood =-139.20399

Three months preventive treatment with rifampicin and isoniazid was prescribed for 61% of children with a positive QFT. Among first and second generation immigrant and Western-born children with a positive QFT, the percentages were 83%, 80% and 19% respectively. The QFT-positive children not treated were enrolled in a three year follow-up program. None of the QFT-negative children were offered preventive treatment and further follow up was terminated for the majority.

## Discussion

In this study, we have shown that only 9% of TST positive school children, including first and second generation immigrants tested positive with the QuantiFERON^®^TB Gold test. QFT positivity was associated with size of TST induration, origin outside Western countries and known exposure to tuberculosis.

### Low prevalence of positive QFT-results

First and second generation immigrants were more likely to have a positive TST reaction. Most of them are already BCG-vaccinated, a factor known to affect the specificity of TST. The assumption that factors other than tuberculosis infection are widely contributing to positive TST reactions among schoolchildren in Norway is supported by this study, as only 9% of TST positive children also had a positive QFT. Thus, QFT seems to be more specific than TST. Sensitivity or differences in the nature of the immune response measured by the two tests should however also be considered. Other studies from low-prevalence countries have reported moderate agreement between the tests, but these studies have included mainly recent contacts or patients with suspected tuberculous disease [6-10,12,14].

TST reactions caused by previous BCG-vaccination or infections with NTM are expected to be moderate and in the range of 6–14 mm [15-17], consistent with our study where 79% of the positive TST reactions occurred within this range. The effect of infant BCG-vaccination on TST is perceived to be minimal, especially more than 10 years after vaccination [18]. Contrary to our results, other studies have reported a significantly better agreement between the tests for unvaccinated than vaccinated groups [8,19]. Different risk profile for tuberculosis exposure between the vaccinated and unvaccinated participants in our study may explain the lack of difference among the groups. Infections from NTM could explain the high number of TST positive children, as these infections are quite frequent in Norway (250 notifications from laboratories every year, unpublished data from the Tuberculosis Register) compared to the 300 notified cases of tuberculosis. They are endemic also in countries with high levels of tuberculosis [20].

Among the children with TST =>15 mm, only 22% had a positive QFT. This discordance is not easily explained by poor specificity of TST since reactions exceeding 15 mm have been interpreted as true tuberculosis infection [15-17]. The sensitivity of QFT for detection of latent infection has been extrapolated from data obtained from patients with active disease and found to be variable (75 – 97%) depending on the study population and design [12,21,22]. Discrepant results between TST =>15 mm and QFT have also been documented also in other studies [20,23,24]. The two tests measure different immune responses. Tuberculin induces a delayed-type hypersensitivity reaction reflecting a memory response, while the blood tests are believed to detect cellular IFN-γ release reflecting an effector response to an ongoing infection. Thus, IFN-γ release assays may detect recent and persistent infection whereas TST react to previous infections [7,12,25]. However, this may be of less relevance in our study as the population studied is very young. The findings illustrate the need for follow-up studies to better clarify which criteria to be considered to diagnose latent tuberculosis and possibly also guide the decision to offer preventive treatment. The tests' potential ability to identify those at risk for progression to disease is of great interest. Until July 2008 none of the participants have been notified with tuberculosis disease in Norway.

None of the seven Western-born QFT positive cases from the same coastal area reported exposure to tuberculosis and careful source case investigations have revealed no links or index case. The incidence rate of tuberculosis in this region was lower than the national average, 4.3 per 100 000 population in 2006 [1], no recent outbreaks have been reported from the area and no case of active tuberculosis has been reported from this school for decades. There is no explanation why this city and school is overrepresented. Despite the high specificity of the QFT, we cannot rule out false positive results caused by one of the species that share the antigens used in the QFT assay with the *M. tuberculosis*-complex [12,26]. The seven QFT-positive children were all healthy with normal chest X-rays, they had no wounds or other symptoms of *M. marinum, M. szulgai*, or *M. kansasii *infections or reported such history. This is a coastal area where children are often in contact with sea water. One of the seven was a sports swimmer, one had an aquarium, two were frequently fishing, but none had clinical findings or reported history of symptoms compatible with *M. marinum *infection, which is normally fairly easy to diagnose.

In this study, QFT testing followed a tuberculin skin test. Leyten found that the specificity of QFT was not affected by prior TST administration [27]. Even though the question is not completely resolved there is no evidence that TST affects the QFT result. Having a QFT following a TST is also in agreement with the European Consensus Report [28].

### Predictors of positive QFT

Origin of the child and known exposure to tuberculosis were predictors of a positive QFT. We assume that most first generation immigrant children with latent infection were infected in their home country prior to arrival in Norway. This is supported by results from Restricted Fragment Length Polymorphism (RFLP) fingerprinting routinely performed on all *M. tuberculosis *isolates in Norway since 1994, indicating that most tuberculosis cases are due to importation of new strains rather than transmission within Norway [3,4]. Second generation immigrant children have been regarded as a risk group for tuberculosis since the disease is mainly affecting the immigrant population, and these children often travel for extended periods to their parents country of origin and live close to the indigenous population. Over the last five years, 4–5 cases of tuberculosis disease have been reported annually among second generation immigrant children in Norway, compared to approximately one case among Western-born children. In this study history of travel was not associated with QFT positivity for any of the groups, even though a substantial number reported accumulated duration of travel exceeding three months.

The study was based on a well-run, public, school-based programme and enrolment was almost complete. The validity of the TST-results can be debated since a lot of public health nurses were involved in the screening, due to the routine setting in which the study was conducted. This is a limitation of the study. Even though none reported non-standardised administration or reading of TST, this can not be ruled out. A moderate digit preference for different TST cut-offs was detected. We also believe that some schools may not have tested previously BCG-vaccinated children or not referred children with marginally positive TST-results. Thus, we may have missed some TST-positive children.

Even though only few participants reported exposure to an infectious case of tuberculosis, this was, as reported in other studies, significantly related to a positive QFT [6,8,9,19,29]. Cultural concepts of "being exposed" may differ since none of the Western-born children and only one of the second generation children reporting exposure were QFT positive. With only 44 QFT positives in the study group of 511 children, however, we had limited power to detect associations between background variables and QFT positivity. Importantly, we observed a significant association between the increase in TST indurations and the proportion of QFT positive. This is consistent with the expected association between the increase in TST indurations and the proportion of tuberculin reactions caused by *M. tuberculosis *[30]. As positive mitogen control was not available as a part of the assay used in this study, we may theoretically have missed some QFT positives. However, this is not so relevant in this population where all participants were TST positive and immunosuppression is considered to be very rare.

### Reproducibility

It is a concern that it was only possible to reproduce a positive result on the same plasma sample in 72% of the cases. A potential discordance occurring at or near to the cut-off value is probably as expected and is also described in other studies [31,32]. Still, we had some initially moderate and a few strongly positive results which we were not able to reproduce, not in repeat analysis of the same sample or for some, in a new test. We do not know to what extent repeated testing of initially negative results also would show inconsistency and how that could have affected the outcome of our study. Among the eight participants who consented to a new test the time between collections of the two samples differed, but none reported any risk of exposure during the period.

The risk of tuberculosis infection and recent exposure is generally low in this study group. In a previous study we have performed among asylum seekers arriving from high incidence countries, we observed a lower proportion (3,7%) of non-conclusive QFT results [33]. This may indicate that the reproducibility of QFT testing can differ between study groups representing differences in incidence and/or levels of positive QFT results. In general discordant results are difficult to interpret and need to be evaluated in larger studies designed for this purpose.

It will not be a standard procedure in a normal screening setting to confirm all positive results by re-analysis of the same plasma sample. This was however done in this study since we assumed that a positive QFT result potentially would increase the likelihood of preventive treatment. QFT is a new test with few data from screening among children and we felt that retesting positive samples would provide more valid results. We assessed the potential impact of this modification in the study by including the 16 participants with an initial non-conclusive first QFT result as QFT-positives in an additional analysis (data not shown). While the numbers and proportions of QFT positives increased, most among Western-born children, there were no significant changes of proportions of positive tests by gender, origin or BCG-scar. In the multivariate analysis exposure was no longer a predictor for a positive QFT while origin remained significant. Thus, including the 16 cases as positives would not materially change the interpretation of the results or the main conclusions of the study.

### Implications for the screening programme

The study suggests that the prevalence of latent infection among 9th grade school children in Norway is very low and that TST screening results in many false positive reactions. Preventive treatment was almost exclusively given to children with immigrant background. Few Western-born children with positive TST and QFT were offered preventive treatment. This probably reflects; (1) no identified source of exposure, (2) an assessment of low risk of subsequent development to active disease and (3) parents not accepting preventive treatment when offered. These results are relevant for the discussion on the usefulness of the screening since identification of children with latent infection for preventive treatment has been one of the aims of the programme. Thus, the current screening programme may not be justified and cost studies are needed. We believe that the few children where infection with *M. tuberculosis *is most likely, may be identified by improved immigrant entry-screening and testing of contacts of cases with infectious tuberculosis. The usefulness of TST as pre-screening before routine BCG vaccination needs to be carefully evaluated in conjunction with the current evaluation of the BCG vaccination programme as a whole.

## Conclusion

Our results indicate that factors other than tuberculosis, such as NTM infection or previous BCG-vaccination, are widely contributing to positive TST results among children in Norway. The poor agreement between TST and QFT tests is therefore mainly explained by the increased specificity of the QFT. Latent infection seems to be almost absent among Western-born TST positive children, including second generation immigrants, while first generation immigrant children are at increased risk. First generation immigrant children are already targeted by entry screening and improved programme performance of this screening is a better control measure in a low prevalence country. The study supports the need to evaluate the current national screening and BCG-vaccination programme in Norway.

## Competing interests

IH has been paid by Astra Zeneca for holding a lecture on mandatory screening of tuberculosis infection in health care services.

The remaining authors declare that they have no competing interests.

## Authors' contributions

BAW has been responsible for study design, planning, data collection, data analysis and interpretation of data and the writing process, FO has been responsible for the laboratory components of the study and has made substantial contributions to the writing process, TM contributed to planning of the study, interpretation of data and input in the writing process, GEK carried out laboratory work and contributed to interpretation of data, AMDR, INL and IH contributed in planning of the study, data collection, data analysis and the writing process and EH contributed to planning of the study, data interpretation and analyses of results as well as giving substantial input in the writing process. All authors read and approved the final manuscript.

## Pre-publication history

The pre-publication history for this paper can be accessed here:


